# Increased food energy supply as a major driver of the obesity epidemic: a global analysis

**DOI:** 10.2471/BLT.14.150565

**Published:** 2015-07-01

**Authors:** Stefanie Vandevijvere, Carson C Chow, Kevin D Hall, Elaine Umali, Boyd A Swinburn

**Affiliations:** a School of Population Health, University of Auckland, 261 Morrin Road, Auckland, New Zealand.; b Laboratory of Biological Modeling, National Institutes of Health, Bethesda, United States of America.

## Abstract

**Objective:**

We investigated associations between changes in national food energy supply and in average population body weight.

**Methods:**

We collected data from 24 high-, 27 middle- and 18 low-income countries on the average measured body weight from global databases, national health and nutrition survey reports and peer-reviewed papers. Changes in average body weight were derived from study pairs that were at least four years apart (various years, 1971–2010). Selected study pairs were considered to be representative of an adolescent or adult population, at national or subnational scale. Food energy supply data were retrieved from the Food and Agriculture Organization of the United Nations food balance sheets. We estimated the population energy requirements at survey time points using Institute of Medicine equations. Finally, we estimated the change in energy intake that could theoretically account for the observed change in average body weight using an experimentally-validated model.

**Findings:**

In 56 countries, an increase in food energy supply was associated with an increase in average body weight. In 45 countries, the increase in food energy supply was higher than the model-predicted increase in energy intake. The association between change in food energy supply and change in body weight was statistically significant overall and for high-income countries (*P* < 0.001).

**Conclusion:**

The findings suggest that increases in food energy supply are sufficient to explain increases in average population body weight, especially in high-income countries. Policy efforts are needed to improve the healthiness of food systems and environments to reduce global obesity.

## Résumé

**Objectif:**

Nous avons enquêté sur les associations entre l’évolution des disponibilités énergétiques alimentaires nationales et celle du poids moyen des populations.

**Méthodes:**

Nous avons collecté les données de 24 pays à revenu élevé, 27 pays à revenu intermédiaire et 18 pays à faible revenu concernant le poids moyen de la population, tel que renseigné dans les bases de données mondiales, les rapports d'enquêtes nationales sur la santé et la nutrition et des articles examinés par comité de lecture. Les changements de poids ont été déterminés par des paires d'études espacées d’au moins quatre années d'intervalle (diverses années sur la période 1971 à 2010). Les paires d'études sélectionnées sont considérées comme représentatives d'une population adolescente ou adulte, à des échelles nationales ou sous-nationales. Les données relatives aux disponibilités énergétiques alimentaires ont été extraites des bilans des disponibilités alimentaires de l'Organisation des Nations Unies pour l'alimentation et l'agriculture (FAO). Nous avons estimé les besoins caloriques des populations aux moments de réalisation des enquêtes en utilisant les équations de l'Institute of Medicine (IOM). Enfin, à l'aide d'un modèle validé expérimentalement, nous avons estimé le changement de l'apport calorique qui pourrait correspondre théoriquement aux changements observés du poids moyen.

**Résultats:**

Dans 56 pays, une augmentation de la disponibilité énergétique alimentaire a été associée à une augmentation du poids moyen. Dans 45 pays, l'augmentation de la disponibilité énergétique alimentaire a été plus importante que l'augmentation de l'apport calorique déduit du modèle. L'association entre l'évolution de la disponibilité énergétique alimentaire et le changement de poids a été statistiquement significative, de manière générale et dans les pays à revenu élevé (P < 0,001).

**Conclusion:**

Ces résultats suggèrent que l'accroissement de la disponibilité énergétique alimentaire suffit à expliquer les augmentations du poids moyen de la population, notamment dans les pays à revenu élevé. Des efforts politiques sont nécessaires pour obtenir des environnements et systèmes alimentaires plus sains afin de réduire l'obésité à l'échelle mondiale.

## Resumen

**Objetivo:**

Se investigó la relación entre los cambios en el suministro nacional de energía alimentaria y el peso corporal medio de la población.

**Métodos:**

Se recopilaron datos de 24 países de ingresos altos, 27 de ingresos medios y 18 de ingresos bajos en relación con el peso corporal medio a partir de bases de datos internacionales, informes de estudios sobre salud y nutrición nacional y estudios revisados por homólogos. Los cambios en el peso corporal medio se obtuvieron a partir de estudios por pares realizados con una diferencia de al menos cuatro años (distintos años, 1971-2010). Los estudios seleccionados se consideraron representativos de una población adolescente o adulta, a nivel nacional o subnacional. Los datos de suministro de energía alimentaria se obtuvieron de los balances sobre alimentación de la Organización de las Naciones Unidas para la Agricultura y la Alimentación. Se calcularon los requisitos energéticos de la población en el momento de la realización de los estudios utilizando ecuaciones del Instituto de Medicina. Por último, se calculó el cambio en la ingesta de energía que, en teoría, podría explicar el cambio observado en el peso corporal medio mediante el uso de un modelo experimentalmente validado.

**Resultados:**

En 56 países, se relacionó un aumento del suministro de energía alimentaria con un aumento del peso corporal medio. En 45 países, el aumento del suministro de energía alimentaria fue mayor que el aumento de ingesta energética previsto en el modelo. La relación entre el cambio en el suministro energético alimentario y el cambio en el peso corporal fue estadísticamente significativo en general y en países de ingresos altos (*P* < 0,001).

**Conclusión:**

Los resultados sugieren que el incremento en el suministro de energía alimentaria basta para explicar el aumento del peso corporal medio de la población, en especial en países de ingresos altos. Es necesario que los políticos se esfuercen por mejorar la salubridad de los entornos y sistemas alimentarios para reducir la obesidad mundial

## ملخص

**الغرض:**

قمنا بتقصي الارتباطات ما بين التغيرات في إمدادات الطاقة الغذائية الوطنية وفي متوسط وزن الجسم للسكان.

**الطريقة:**

لقد جمعنا بيانات من 24 بلدًا من البلدان عالية الدخل و27 بلدًا متوسط الدخل و18 بلدًا منخفض الدخل حول متوسط وزن الجسم من قواعد البيانات العالمية، وتقارير المسح الوطنية للصحة والتغذية، والدراسات التي خضعت لاستعراضات نديّة. وكانت التغيرات في متوسط وزن الجسم مستمدة من أزواج الدراسة التي تباعدت لمدة أربع سنوات على الأقل (سنوات عديدة، في الفترة ما بين عام 1971 إلى عام 2010). وتم اعتبار أزواج الدراسة المختارة لتكون ممثلة للقطاعات السكانية في سن المراهقة أو البالغين على الصعيد الوطني أو دون الوطني. تم الحصول على بيانات إمدادات الطاقة الغذائية من الميزانيات التموينية لمنظمة الأغذية والزراعة التابعة للأمم المتحدة. وقد قدّرنا متطلبات الطاقة للسكان في فترات زمنية للمسح باستخدام المعادلات الخاصة بمعهد الطب. وأخيرًا، قدّرنا التغير في مدخول الطاقة الذي قد يكون مسؤولاً من الناحية النظرية عن التغير الخاضع للرصد في متوسط وزن الجسم باستخدام نموذج تم التحقق من مصداقيته من واقع التجارب.

**النتائج:**

في 56 بلدًا، ارتبطت الزيادة في إمدادات الطاقة الغذائية بزيادة في متوسط وزن الجسم. في 45 بلدًا، كانت الزيادة في إمدادات الطاقة الغذائية أعلى من زيادة مدخول الطاقة الغذائية التي تنبأ بها النموذج. وكان الارتباط بين التغير في إمدادات الطاقة الغذائية والتغير في وزن الجسم ملموسًا من الناحية الإحصائية بشكل إجمالي وبالنسبة للبلدان عالية الدخل ( *الاحتمال * > ‏0.001).

**الاستنتاج:**

تشير النتائج إلى أن الزيادات في إمدادات الطاقة الغذائية كافية لشرح الزيادات في متوسط وزن الجسم للسكان، ولا سيما في البلدان عالية الدخل. وهناك حاجة إلى بذل جهود من خلال السياسات لتحسين سلامة النظم والبيئات الغذائية لخفض معدلات السمنة العالمية.

## 摘要

**目的:**

我们调查了全国食品能量供给和人口平均体重之间的变化联系。

**方法:**

我们从 18 个低收入国家的平均体重数据。平均体重的变化来源于最少相差 4 年的两组研究（不同年份，1971–2010 年）。所选的两组研究被认为是代表了国家或地方范围内青少年或成年人口的情况。食品能量供给的数据是从联合国粮食和农业组织的食品平衡表检索而得。我们采用医学研究所的方程式估计了在调查时间点的人口能量需求。最后，我们通过模型估计了能够在理论上对平均体重观察得到的变化产生影响的能量摄入的变化情况。

**结果:**

在 56 个国家中，食品能量供给的增加与平均体重的增加相互联系。与通过模型预测的能量摄入相比， 45 个国家中食品能量供给的增加更高。能量供给变化与体重变化之间的联系在高收入国家中从整体上存在统计学上的显著差异 (*P* < 0.001)。

**结论:**

结果表明食品能量供给的增加足以构成平均人口体重增加的原因，尤其是在高收入国家。我们还需要加大政策力度，改善食品系统和环境的健康，从而降低全球肥胖率。

## Резюме

**Цель:**

Исследование взаимосвязи между изменениями в калорийности продуктов питания и средней массы тела у населения.

**Методы:**

Были собраны данные о средней массе тела у населения на основании глобальных баз данных, рецензируемых документов и отчетов о национальном исследовании состояния здоровья и питания населения. Анализ проводился по данным из 24 стран с высоким уровнем дохода, 27 стран со средним уровнем дохода и 18 стран с низким уровнем дохода. Информация об изменении средней массы тела была получена в ходе исследования пар, возраст которых отличался как минимум на четыре года (различные года в период с 1971 по 2010 г.). Выбранные для исследования пары считались характерными для подросткового и взрослого населения на национальном или субнациональном уровне. Данные о калорийности пищи были получены из продовольственного баланса Продовольственной и сельскохозяйственной Организации Объединенных Наций. Энергетические потребности населения оценивались на момент исследования с использованием уравнений Института медицины. Изменения в калорийности потребляемой пищи, которые теоретически могли служить причиной для наблюдаемого изменения средней массы тела, оценивались с использованием экспериментально проверенной модели.

**Результаты:**

В 56 странах повышение калорийности пищи было связано с увеличением средней массы тела. В 45 странах повышение калорийности пищи превышало значение, прогнозируемое моделью. Взаимосвязь между изменением калорийности пищи и изменением массы тела была статистически значима повсеместно, в том числе и для стран с высоким уровнем дохода (*P* < 0,001).

**Вывод:**

Результаты исследований свидетельствуют о том, что повышение калорийности пищи с достаточной вероятностью объясняет увеличение средней массы тела у населения, особенно в странах с высоким уровнем дохода. Для снижения уровня ожирения в мировом масштабе нужно предпринимать стратегические меры, направленные на создание пищевого окружения и систем питания, в большей мере способствующих здоровому уровню жизни.

## Introduction

Overweight and obesity have become major global public health problems. Worldwide, the proportion of adults with a body mass index (BMI) of 25 kg/m^2^ or greater increased from 28.8% to 36.9% in men, and from 29.8% to 38.0% in women between 1980 and 2013.^1^ Urgent action from governments and the food industry is needed to curb the epidemic.^2^ Action needs to be directed at the main drivers of the epidemic to meet the global target of halting the rise in obesity by 2025.^3^

The drivers of the obesity epidemic have been much debated.^4^^–^^7^ An increased food energy supply and the globalization of the food supply, increasing the availability of obesogenic ultra-processed foods, are arguments for a predominant food system driver^5^ of population weight gain. Increasing motorization and mechanization, time spent in front of small screens and a decrease in transport and occupational physical activity, point to reducing physical activity as a predominant driver^6^^,^^8^ of the obesity epidemic.

A model used to predict body-weight gain, assuming no change in physical activity, follows the simple rule that a sustained increase in energy intake of 100 kJ per day leads to a predicted increase of 1 kg body weight on average, with half of the weight gain being achieved in about one year and 95% in about three years.^9^ According to this model, the oversupply of food energy is sufficient to drive the increase in energy intake and increases in body weight observed in the United Kingdom of Great Britain and Northern Ireland and the United States of America.^9^^–^^11^ This is despite the fact that, in the United States, food waste has increased by approximately 50% since 1974, reaching about 5800 kJ per person per day in 2003.^12^ Here we test the hypothesis that an increase in food energy supply is sufficient to explain increasing population body weight, using data from 24 high-income, 27 middle-income and 18 low-income countries.

## Methods

### Food energy supply

Food balance sheets of the Food and Agriculture Organization of the United Nations (FAO) estimate the food supply of countries, by balancing local production, country-wide stocks and imports with exports, agricultural use for livestock, seed and some components of waste. Waste on the farm, during distribution and processing, as well as technical losses due to transformation of primary commodities into processed products are usually taken into account. However, losses of edible food, e.g. during storage, preparation and cooking, as plate-waste or domestic animal feed, or thrown away, are not considered. The data are expressed as the annual per capita supply of each food item available for human consumption.^13^ The FAO’s database contains national level data from 1961 to 2010 for 183 countries. For each country, data on food energy supply were extracted to match the time periods of data on adult body weight.

### Measured body weight

Three major strategies were used to collect data on measured average adult body weight. First, an electronic search of major databases on obesity prevalence and BMI was performed, including the World Health Organization’s (WHO) global infobase,^14^ WHO’s global database on BMI,^15^ the International Association for the Study of Obesity (now World Obesity Federation) database^16^ and the Organisation for Economic Co-operation and Development’s health data.^17^ As these databases only included data on obesity rates or mean BMI, the original sources of the data were searched. Second, data on average measured body weight were gathered from reports of national health and nutrition surveys in various countries. The WHO MONICA project^18^ and WHO STEPwise approach to surveillance (STEPS) country reports^19^ included anthropometric measures for male and female adult samples. We also calculated body weight for women of child-bearing age using mean BMI and height data from Demographic and Health Surveys.^20^ Third, an electronic search of Medline was conducted. For each country, a separate search was performed using the following keywords: “obesity”, “weight”, “anthropometric”, “BMI”, “health survey” and “national survey” (using the Boolean operator OR). Finally, specific national health and/or nutrition surveys identified by some of the above sources were electronically searched.

Studies fulfilling the following criteria were extracted: (i) weight was measured after 1961 and again before 2010 (to match the FAO food balance sheet data); (ii) the study samples were representative of a national or subnational adolescent or adult population; (iii) the survey method was comparable with previous or future surveys conducted in the country; (iv) the year in which each survey was conducted could be identified; at least four years elapsed between the two surveys; and (v) FAO food supply data were available for the relevant period.

If there were more than two eligible studies from a country, the surveys which we judged to be the best quality were included. Criteria for estimating study quality included national representativeness, sample size and length of time between surveys.

### Demographic data

Demographic data (total population, by age and sex) were retrieved from the United Nations Department of Economic and Social Affairs.^21^ Average female and male height at survey time points were derived from http://www.averageheight.co/. For 13 countries, data were not available and average height data from a neighbouring country were used for calculating energy requirements.

### Data analysis

Three types of analysis were performed. First, we compared the changes in food energy supply with changes in average body weight over time for each country. Second, estimates of population energy requirements at survey time points were performed for each country using Institute of Medicine equations.^22^ Low active physical activity levels (1.4 ≤ PAL <1.6) were assumed for high- and upper-middle-income countries. Active physical activity levels (1.6 ≤ PAL <1.9) were used for all other countries. Finally, we used a physiologically-based, experimentally-validated predictive energy intake body-weight model, to estimate the change in average population energy intake that would be required to account for the observed change in average body weight.^9^

## Results

In total, 83 countries had at least two surveys with data on measured body weight; 24 countries had more than two surveys at different time points. We excluded countries where the period between surveys was less than four years (eight countries), survey populations were not comparable in terms of area representativeness (eight countries) or FAO food supply data for the country were not available (three countries). Survey pairs from 69 countries were included. Of those, 36 survey pairs included data for women of childbearing age only. One survey pair (Saudi Arabia) included data for men only. Data from 24 high-income, 27 middle-income and 18 low-income countries were included. The average period between the surveys was 12 years (range 4–37 years; Table 1). At the time of the initial survey, food energy supply was greater than the average energy requirements in 52 countries. For 37 of these countries, this excess food energy supply was more than 2000 kJ/day (Table 1).

**Table 1:** Countries and surveys included in a global analysis of food energy supply and body weight, 1971–2010

Country	Income level of country	Year				Age range, years		Food energy supply, kJ/day		
		First survey	Second survey	Survey 1	Survey 2	First survey	Second survey	First survey	Change	Excess at the first survey
Algeria	Upper-MIC	1986	2003	Cross-sectional survey	STEPS Survey	16–65	25–64	11 385	1 464	2 958
Australia	HIC	1995	2007	National Nutrition Survey	National Health Survey	≥ 18	≥ 18	12 929	594	2 987
Bangladesh	LIC	1996	2007	National Demographic Health Survey	National Demographic Health Survey	15–49	15–49	8 849	1 423	506
Barbados	HIC	1995	2000	ICSHIB Study	Food Consumption and Anthropometric Survey	≥ 25	18–96	11 996	−146	2 414
Belgium	HIC	1986	1991	WHO MONICA	WHO MONICA	25–34	25–34	14 439	515	4 008
Benin	LIC	1996	2001	National Demographic Health Survey	National Demographic Health Survey	15–49	15–49	9 929	54	715
Bolivia (Plurinational State of)	Lower-MIC	1994	2008	National Demographic Health Survey	National Demographic Health Survey	15–49	15–49	8 376	544	−285
Burkina Faso	LIC	1993	1998	National Demographic Health Survey	National Demographic Health Survey	15–49	15–49	10 092	−109	728
Cambodia	LIC	2000	2010	National Demographic Health Survey	STEPS Survey	15–49	25–64	8 908	1 059	197
Cameroon	Lower-MIC	1998	2004	National Demographic Health Survey	National Demographic Health Survey	15–49	15–49	8 870	774	−649
Canada	HIC	1971	2008	Nutrition Canada Survey	Canadian Community Health Survey	20–69	≥ 18	12 159	2 339	2 636
Chad	LIC	1996	2004	National Demographic Health Survey	National Demographic Health Survey	15–49	15–49	7 740	895	−1 665
Chile	HIC	2003	2009	National Health Survey	National Health Survey	≥ 17	≥ 15	12 067	100	2 665
China	Upper-MIC	1991	2000	China Health and Nutrition Survey	Cross-sectional survey	20–45	35–74	10 447	1 548	1 996
Colombia	Upper-MIC	1995	2005	National Demographic Health Survey	National Demographic Health Survey	15–49	15–49	10 837	188	2 510
Czech Republic	HIC	1993	2002	Health Status of the Czech Population Survey	Health Status of the Czech Population Survey	15–75	15–75	12 719	833	2 653
Denmark	HIC	1983	1991	WHO MONICA	WHO MONICA	25–64	25–64	12 740	862	2 795
Dominican Republic	Upper-MIC	1991	1996	National Demographic Health Survey	National Demographic Health Survey	15–49	15–49	9 025	301	749
Egypt	Lower-MIC	1992	2005	National Demographic Health Survey	National Demographic Health Survey	15–49	15–49	13 142	741	3 284
Eritrea	LIC	1995	2003	National Demographic Health Survey	National Demographic Health Survey	15–49	15–49	6 569	−63	−2 272
Ethiopia	LIC	2000	2005	National Demographic Health Survey	National Demographic Health Survey	15–49	15–49	7 565	761	−1 343
Fiji	Upper-MIC	1980	2004	National Food and Nutrition Survey	STEPS Survey (National Nutrition Survey)	18–55	18–55	10 372	2 301	88
Finland	HIC	1987	1997	Cross-sectional population survey	Cross-sectional population survey	25–64	25–64	12 318	849	2 289
France	HIC	1986	2009	WHO MONICA	National Epidemiological Survey	35–64	≥ 18	14 707	67	5 067
Gabon	Upper-MIC	2000	2009	National Demographic Health Survey	STEPS Survey	15–49	15–64	11 234	251	2 653
Germany	HIC	1983	2009	WHO MONICA	Microcensus – Health Questions	25–64	≥ 18	14 267	582	4 305
Ghana	Lower-MIC	1993	2003	National Demographic Health Survey	National Demographic Health Survey	15–49	15–49	9 468	1 289	213
Haiti	LIC	1994	2005	National Demographic Health Survey	National Demographic Health Survey	15–49	15–49	7 163	703	−1 929
Hungary	Upper-MIC	1982	1987	WHO MONICA	WHO MONICA	25–64	25–64	14 836	753	4 640
Iceland	HIC	1983	1993	WHO MONICA	WHO MONICA	25–64	25–64	13 334	−343	2 757
India	Lower-MIC	1998	2007	National Demographic Health Survey	STEPS Survey	15–49	15–64	9 657	113	715
Indonesia	Lower-MIC	1983	2001	Cross-sectional survey	STEPS Survey	15–49	15–65	9 615	276	1 423
Iran (Islamic Republic of)	Upper-MIC	2004	2009	STEPS Survey	STEPS Survey	15–65	15–64	13 129	25	3 540
Ireland	HIC	1985	2009	Cross-sectional survey	National Adult Nutrition Survey	35–64	18–64	14 966	109	5 209
Israel	HIC	1985	2000	WHO MONICA	National Health and Nutrition Survey	25–64	25–64	13 979	728	4 284
Italy	HIC	1983	1993	WHO MONICA	WHO MONICA	25–64	25–64	14 493	71	4 749
Jordan	Upper-MIC	1997	2002	Cross-sectional survey	National Demographic Health Survey	≥ 25	15–49	11 355	720	2 778
Kazakhstan	Upper-MIC	1995	1999	National Demographic Health Survey	National Demographic Health Survey	15–49	15–49	13 117	−3 778	4 448
Kenya	LIC	1993	2003	National Demographic Health Survey	National Demographic Health Survey	15–49	15–49	7 954	444	−1 318
Lebanon	Upper-MIC	1997	2009	National cross-sectional survey	National cross-sectional survey	≥ 20	≥ 20	12 924	268	2 983
Madagascar	LIC	1997	2005	National Demographic Health Survey	STEPS Survey	15–49	25–64	8 732	155	−67
Malawi	LIC	1983	2009	Cross-sectional survey	STEPS Survey	≥ 15	25–64	9 012	686	−690
Malaysia	Upper-MIC	1996	2005	National Health & Morbidity Survey	STEPS Survey	≥ 20	25–64	12 355	−481	3 745
Mali	LIC	1995	2006	National Demographic Health Survey	National Demographic Health Survey	15–49	15–49	9 021	1 155	−322
Malta	HIC	1984	2006	WHO MONICA	Lifestyle Survey	25–64	18–65	12 711	1 682	3 130
Mauritania	Lower-MIC	2000	2006	National Demographic Health Survey	STEPS Survey	15–49	15–64	11 351	59	1 636
Mongolia	Lower-MIC	2005	2009	STEPS Survey	STEPS Survey	15–64	15–64	9 410	774	−891
Morocco	Lower-MIC	1992	2003	National Demographic Health Survey	National Demographic Health Survey	15–49	15–49	12 117	1 331	2 611
Mozambique	LIC	1997	2003	National Demographic Health Survey	National Demographic Health Survey	15–49	15–49	8 263	247	−728
Nepal	LIC	1996	2007	National Demographic Health Survey	STEPS Survey	15–49	15–64	9 234	674	766
Netherlands	HIC	2000	2009	Health Survey	Health Survey	15–65	15–65	13 389	255	2 941
New Zealand	HIC	1982	2009	WHO MONICA	NZ Adult Nutrition Survey	35–64	15–71	12 878	389	3 234
Niger	LIC	1992	2006	National Demographic Health Survey	National Demographic Health Survey	15–49	15–49	8 142	1 598	−1 025
Nigeria	Lower-MIC	1999	2003	National Demographic Health Survey	National Demographic Health Survey	15–49	15–49	11 109	−134	1 741
Norway	HIC	1990	2001	Prospective population-based survey	Prospective population-based survey	≥ 20	20–79	13 196	992	3 280
Peru	Upper-MIC	1991	2009	National Demographic Health Survey	National Demographic Health Survey	15–49	15–49	9 075	1 653	874
Poland	HIC	1983	1992	WHO MONICA	WHO MONICA	35–64	35–64	14 046	243	4 339
Rwanda	LIC	2000	2005	National Demographic Health Survey	National Demographic Health Survey	15–49	15–49	7 812	674	−1 385
Saudi Arabia	HIC	1996	2004	Cross-sectional survey	STEPS Survey	≥ 19	25–64	12 247	519	1 448
Senegal	Lower-MIC	1992	2005	National Demographic Health Survey	National Demographic Health Survey	15–49	15–49	9 427	506	−155
South Africa	Upper-MIC	1998	2003	National Demographic Health Survey	National Demographic Health Survey	15–65	15–65	11 929	397	2 243
Sweden	HIC	1985	2001	WHO MONICA	INTERGENE Project	25–64	25–64	12 456	636	2 703
Switzerland	HIC	1985	1994	WHO MONICA	WHO MONICA	35–64	25–64	14 242	−310	4 590
Togo	LIC	1998	2010	National Demographic Health Survey	STEPS Survey	15–49	15–64	9 150	736	−469
Turkey	Upper-MIC	1993	2003	National Demographic Health Survey	National Demographic Health Survey	15–49	15–49	15 531	−602	7 251
United Kingdom	HIC	1993	2009	Health Survey for England	Health Survey for England	≥ 16	≥ 16	13 468	891	3 724
United States	HIC	1972	2004	National Health and Nutrition Examination Survey	National Health and Nutrition Examination Survey	20–74	20–74	12 770	3 213	2 979
Uzbekistan	Lower-MIC	1996	2002	National Demographic Health Survey	Health Examination Survey	15–49	15–49	12 242	−2 615	2 803
Zimbabwe	LIC	1994	1999	National Demographic Health Survey	National Demographic Health Survey	15–49	15–49	8 037	280	−1 343

LIC: low-income country; Lower-MIC: lower-middle-income country; HIC: high-income country; ICSHIB: the International Comparative Study of Hypertension in Blacks; Upper-MIC: upper-middle-income country; WHO: World Health Organization.

Note: Estimations of population energy requirements were performed for each country using the Institute of Medicine equations for males and females.^22^ Energy excess was calculated by subtracting energy requirements at the first survey from the energy supply at the same survey.

**Fig. 1:**
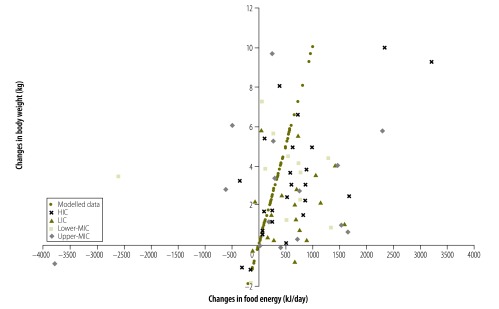
Change in food energy supply and change in average body weight for 69 countries, 1971–2010

LIC: low-income countries; Lower-MIC: lower-middle-income countries; HIC: high-income countris; Upper-MIC: upper-middle-income countries.

Note: The dots representing the modelled data are the estimated change in energy intake required to account for the change in average body weight of the population.^9^

**Fig. 2:**
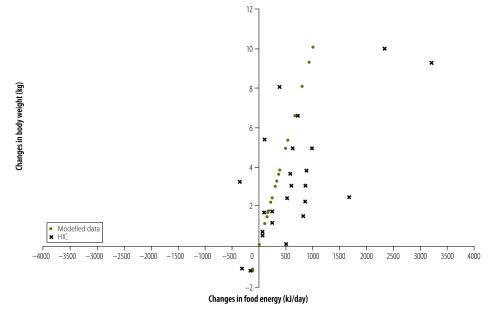
Change in food energy supply and change in average body weight for 24 high-income countries, 1971–2009

**Fig. 3:**
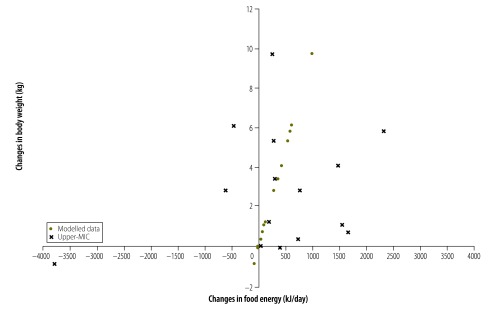
Change in food energy supply and change in average body weight for 15 upper-middle-income countries, 1980–2009

**Fig. 4:**
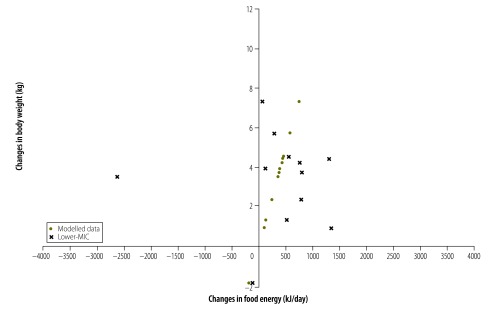
Change in food energy supply and change in average body weight for 12 lower-middle-income countries, 1983–2009

**Fig. 5:**
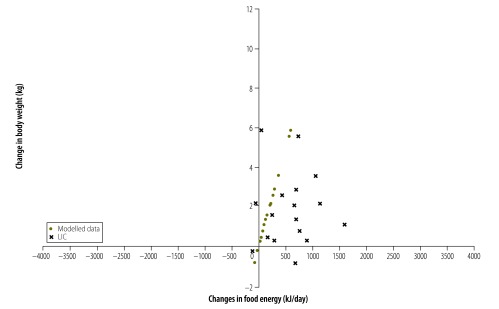
Change in food energy supply and change in average body weight for 18 low-income countries, 1983–2009

For 56 countries (81%) both food energy supply and body weight increased between the survey pairs. For 45 of these countries (80%) the increase in food energy supply was more than sufficient to explain the increase in average body weight. This is shown in Fig. 1 with 56/69 countries being in the top right quadrant and 45/56 being to the right of the model-predicted change in energy intake needed to produce the increase in mean body weight for that country. This same pattern was observed for countries of all income levels (Fig. 2, Fig. 3, Fig. 4 and Fig. 5). For 11 countries (Benin, Chile, the Dominican Republic, Gabon, India, Indonesia, Ireland, Italy, Lebanon, Mauritania and New Zealand) in the top right quadrant, the increase in food energy supply was insufficient to account for the observed increase in weight (Fig. 1).

HIC: high-income countries.

Note: The dots representing the modelled data are the estimated change in energy intake required to account for the change in average body weight of the population.^9^

Upper-MIC: upper-middle-income countries.

Note: The dots representing the modelled data are the estimated change in energy intake required to account for the change in average body weight of the population.^9^

Lower-MIC: lower-middle-income countries.

Note: The dots representing the modelled data are the estimated change in energy intake required to account for the change in average body weight of the population.^9^

LIC: low-income countries.

Note: The dots representing the modelled data are the estimated change in energy intake required to account for the change in average body weight of the population.^9^

Five countries (Barbados, Burkina Faso, Kazakhstan, Nigeria and Switzerland) experienced reductions in both food energy supply and average body weight. For Kazakhstan the food energy supply decreased by 3778 kJ/day, from 13 117 kJ/day to 9339 kJ/day over a four year period (Table 1), accompanied by a decrease in average body weight of 0.9 kg. For the four other countries, decreases in food energy supply were much more modest (100–300 kJ/day; Table 1).

For five other countries (Eritrea, Iceland, Malaysia, Turkey and Uzbekistan), discordant changes were observed with reductions in food energy supply over the same period as increases in average body weight. The decrease in food energy supply was highest for Uzbekistan (2615 kJ/day) and lowest for Eritrea (63 kJ/day; Table 1). Apart from Eritrea, food energy supply at baseline for those five countries was relatively high (ranging from 12 242 to 15 531 kJ/day) and higher than the values of at least half of the other countries included in this study. In addition, excess food energy supply at baseline was high for those five countries (2757–7251 kJ/day; Table 1).

For three countries (the Islamic Republic of Iran, Rwanda and South Africa) there were discordant changes in the other direction with increases in food energy supply over the same period as reductions in average body weight. However, for two of those countries, the change in average weight was small (a reduction of 5 g for the Islamic Republic of Iran and 100 g for South Africa). In Rwanda, the reduction in weight was 800 g while the food energy supply over the same time period increased by 674 kJ/day (Table 1).

The correlation between the change in food energy supply and change in average body weight was significant (*P* = 0.011). When stratifying by type of country, associations were significant for high-income countries (*P* < 0.001), but not for other country groups.

## Discussion

For most of the countries included in this study, the change in per capita food energy supply was greater than the change in food energy intake theoretically required to explain the observed change in average body weight. The associations between changes in food energy supply and average population body weight were significant overall and for high-income countries. This suggests that, in high-income countries, a growing and excessive food supply is contributing to higher energy intake, as well as to increasing food waste.^12^

Other factors, such as a decrease in physical activity, may also lead to an increase in body weight and could occur simultaneously with an increase in food energy supply. It has been shown that among 3.7 million participants in the United States at the county level, increased physical activity has only a very small impact on obesity prevalence.^23^ It is likely that in some countries, such as China, the impact of reduced physical activity on obesity is more important.^24^^,^^25^ A reduction in physical activity with no compensatory drop in energy intake will cause weight gain until sufficient weight is gained to create energy balance (through both an increased resting metabolic rate and increased energy required to move the larger body).

Researchers have suggested additional contributing factors for obesity, such as pollutants, infections and changes in the gut microbiota. These factors have an effect on metabolism, body composition and/or energy balance efficiencies. However, more evidence is needed to understand the importance of these factors in weight gain.^26^ Ideally, the cause of obesity in humans would be assessed through randomized controlled trials, where food energy availability is increased randomly and average body weight is then measured. However, such an experiment is not practical, since it is difficult to measure food intake over long time periods and it would require that non-obese subjects be randomly assigned to environments with different food energy supplies.

Our findings suggest that there is an excess of energy available from an increasing national average food energy supply in countries of varying income levels.^9^ Therefore, policy efforts need to focus on reducing population energy intake through improving the healthiness of food systems and environments.^5^^,^^11^^,^^27^ Achieving WHO’s target to halt the rise in obesity by 2025 will require major action by governments and the food industry.^3^ A combination of several policy actions will be needed to significantly improve diets and reduce overconsumption.^2^ These policies include restriction of unhealthy food marketing to children, front-of-pack supplementary nutrition labelling,^28^ food pricing strategies,^29^ improving the quality of foods in schools^30^ and other public sector settings. The impact of trade and investment agreements^31^ and agricultural policies^32^ on domestic food environments should be assessed.

The main strength of this study is the inclusion of nationally representative body weight and food energy supply data for a range of countries and over many years. Weaknesses include the limitations on the measurement of national per capita food energy supply (e.g. losses of edible food during storage, preparation and cooking, as plate-waste or domestic animal feed, and subsistence farming are not taken into account) and the variable quality of energy supply data. In addition, low- and middle-income countries, in different phases of the nutrition transition,^33^^,^^34^ are likely to have poorer data and have higher levels of subsistence farming, which is not included in the FAO food supply data.^13^

The association between changes in food supply and changes in body weight may be confounded by changes in physical activity levels, changes in food waste or changes in the demographic profile of countries. Demographic changes, particularly size, ageing, and racial/ethnic diversification of populations, may contribute to increasing obesity levels.^35^ About half the data sets on weight status used in this study are for women only and thus only represent half of the population. A limitation of the energy-balance model is that it assumes that metabolic physiology and physical activity levels are similar globally. While this is likely to be true for industrialized countries for which accurate data on the relationship between energy expenditure and body weight are available and for which the model has been calibrated, it is not clear how well this assumption applies for developing countries. The model also assumes that population-wide changes in physical activity are negligible over the periods investigated.

In conclusion, in high-income countries, observed increases in body weight over recent decades are associated with increased food energy supply. In addition, increases in food energy supply are sufficient to explain increases in average population weight. Due to the nutrition transition and a potential decrease in physical activity, the same pattern is expected to occur in low- and middle-income countries in the future. Policy efforts should focus on reducing population energy intake through improving the healthiness of food systems and environments.
